# The yield of continuous EEG monitoring in the intensive care unit at a tertiary care hospital in Saudi Arabia: A retrospective study

**DOI:** 10.12688/f1000research.19237.3

**Published:** 2019-10-18

**Authors:** Haythum O. Tayeb

**Affiliations:** 1 Division of Neurology, Department of Internal Medicine, Faculty of Medicine, King Abdulaziz University, Jeddah, Saudi Arabia

**Keywords:** Neurocritical care, EEG, non-convulsive seizures, status epilepticus, Saudi Arabia

## Abstract

**Background:** The practice of continuous EEG monitoring (CEEG) in the intensive care unit (ICU) has spread over the past decade. Building an effective ICU CEEG program demands adequate EEG equipment and human resources. This may not be available in developing healthcare systems. This study sought to shed light on the real-life utility of CEEG at a tertiary healthcare center in the developing healthcare system of Saudi Arabia.

**Methods:** This is a retrospective review of CEEG findings, along with mortality and duration of hospitalization of patients who had CEEG during a 12-month period at the adult ICU at the King Abdulaziz University Hospital (KAUH) in Jeddah, Saudi Arabia.

**Results:** A total of 202 CEEG records were identified. A total of 52 records showed non-convulsive seizures (NCS); 10 clearly fulfilled criteria for non-convulsive status epilepticus. There were 120 patients that had clinical seizures upon presentation. Among them, 36 (30%) had NCS on EEG. The proportion of patients who were deceased at 60 days was higher in patients with NCS than those who didn’t have NCS (42% vs 27%, χ ^2 ^= 4.4, df=2, p=0.03). There was no statistically significant association between having rhythmic or periodic patterns without NCS and mortality at 60 days or length of hospital stay.

**Conclusion:** This retrospective study demonstrates a real-world experience from a tertiary care center in Saudi Arabia, a developing healthcare system. ICU CEEG was found to be effective in detecting potentially harmful subclinical patterns, supporting the need to develop ICU CEEG programs. However, the incurred excesses in morbidity and mortality associated with CEEG patterns were relatively modest. Further studies are needed to delineate how the practice of CEEG may be developed in similar healthcare systems to provide meaningful data to clinicians with regards to patient outcomes.

## Introduction

Continuous electroencephalography (CEEG), the practice of continuously recording an electroencephalogram and a time-synchronized video of the patient, is commonly utilized to monitor critically ill patients with acute brain injury or altered mental status ^1^. CEEG is instrumental in the diagnosis and management of nonconvulsive seizures (NCS) and status epilepticus, detection of cerebral ischemia, prognostication of outcomes after cardiorespiratory arrest, and evaluation of abnormal movements and altered mental status ^1^. The practice of CEEG monitoring of critically ill patients in the intensive care unit (ICU) has been spreading in Europe and North America over the past decade ^1, 2^. Building an effective ICU CEEG program with sufficient quality demands not only adequate EEG equipment but also significant human resources ^2^, including trained electroencephalographers and technologists with enough time to review large amounts of CEEG data ^2^. While this is available in large tertiary care centers in North America and Europe where the practice of CEEG has developed, it may not be available in developing healthcare systems with constrained resources.

This study reviewed data generated from a CEEG program in the adult ICU at a tertiary healthcare center in Saudi Arabia, aiming to shed light on the real-life utility of CEEG in a developing healthcare system outside North America and Europe.

## Methods

### Data gathering

This is a retrospective review of ICU CEEG findings, as well as mortality status and duration of hospitalization of all patients who underwent CEEG monitoring during a 12-month period from September 2016 to August 2017 at the adult ICU at the King Abdulaziz University Hospital (KAUH) in Jeddah, Saudi Arabia. This is an academic, tertiary-care, 600-bed hospital. Its adult ICU is comprised of 30 beds and is divided into medical and surgical divisions. The average APACHE II (Acute Physiology and Chronic Health Evaluation II) score of medical patients admitted to the ICU ranges between 10–30 on average. The scores are routinely calculated but not recorded in the electronic medical records (EMR). ICU physicians or neurologists request CEEG to search for NCS or rhythmic or periodic patterns when critically ill patients have a disturbance in the level of consciousness that is unexplained by apparent underlying neurological or medical conditions. Some of these patients also exhibit paroxysmal motor or non-motor events in association with altered mental status (these are not always consistently recorded in the EMR upon requesting the EEG). During the study period, 120 patients had clinical seizures upon presentation. CEEG is initiated and stopped based on the clinical judgement of the treating physicians. Generally, physicians aim to continue CEEG monitoring for at least 24 hours in patients with altered mental status but may allow discontinuation of CEEG for clinical or practical constraints (e.g. EEG machine availability or the need to relocate a patient to conduct a procedure or test). The electroencephalographer reviews records at least once daily and communicates findings to the treating physicians. If seizures or other CEEG patterns of significance are revealed, physicians often decide to continue or repeat monitoring on subsequent days to guide management. For the purposes of this study, records that continue for several days and those that are within 60 days of each other were considered as one recording. EEGs with a duration that is less than 2 hours were not included in the study as they were considered extended but not long-term studies. An EEG technologist is available during the day time to set up ICU CEEGs. The time from requesting the CEEG to the beginning of the recording varied depending on availability of equipment and technologists. Generally, requests from the ICU are given priority and fulfilled within 24 hours but this is not always feasible. EEG leads are placed using the 10–20 international system of lead placement. CEEGs are digitally recorded, including synchronized video recording of the patient. An epileptologist with fellowship training in CEEG interpretation reviewed the records on daily basis and reported them using the American Clinical Neurophysiology Society (ACNS) ICU EEG consortium proposed nomenclature for ICU EEG reporting, and the Salzburg criteria for non-convulsive status epilepticus ^3^.

### Data analysis

Reports of CEEGs performed in the adult ICU during the study period were retrieved from the hospital’s EMR. The author extracted key data, including background demographics, diagnoses, length of hospital stay, mortality status at 60 days after admission, and the presence of rhythmic and periodic patterns (RPPs) or NCS. Frequencies, percentages, means, standard deviation, and Chi square were performed using the IBM SPSS Statistics for Windows, version 20.0.

### Ethical approval

This study was approved by the Institutional Review Board of KAUH as a retrospective study of anonymized clinical data with waiver of additional patient consent.

## Results

**Figure 1.**
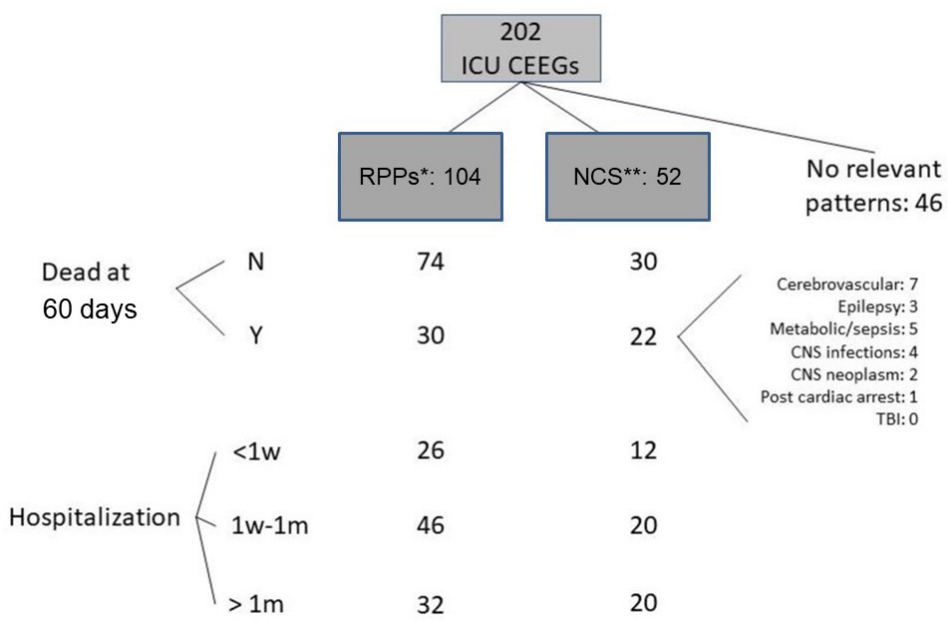
A flow chart showing the frequencies of the CEEG findings, the associated etiologies, and the distribution across outcome categories. * Cases with RPPs without NCS. ** This number includes 34 cases with coexisting RPPs and NCS.

**Figure 2.**
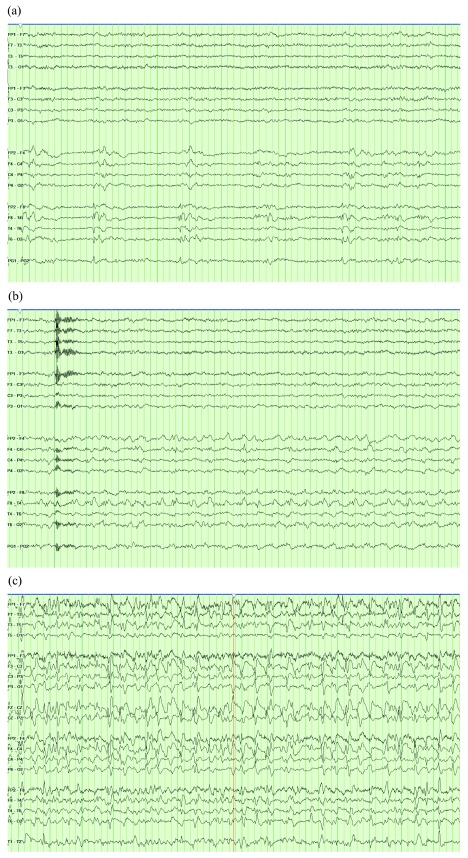
Examples of relevant CEEG patterns from the study sample. EEGs are displayed in a longitudinal bipolar montage with a sensitivity of 7 µV/mm and a time base of 30 mm/second. The high frequency filter is set to 70 Hz and the low frequency filter is set to 1 Hz. ( **a**) Right temporal lateralized periodic discharges (LPDs). ( **b**) The same record showing evolution of the LPDs into 2.5 Hz rhythmic theta activity, fulfilling seizure criteria. ( **c**) Another record showing very long runs of generalized spike and wave activity that recur for most of the recording, fulfilling criteria of non-convulsive status epilepticus.

A total of 202 CEEG records fulfilling the criteria were identified; complete, raw figures are available as *Underlying data* ^4^. There were 116 female patients. The mean age was 53 year (standard deviation=21). The primary diagnosis (as defined by the ICU physicians and entered in the EMR and the EEG requisitions) was epilepsy in 53 patients (26.2%), ischemic stroke in 51 patients (25%), sepsis or metabolic derangement in 40 patients (19.8%), CNS infection in 24 patients (11.9%), intracranial hemorrhage in 10 patients (5%), post-cardiac arrests in 10 patients (5%), brain neoplasm in 8 patients (4%), and traumatic brain injury in 6 patients (3%). The duration of CEEG recording varied, with 48 (24%) recorded for 2–6 hours and 154 (76%) recorded for longer. Figure 1 shows a flowchart demonstrating the frequencies of CEEG findings in the sample and their distribution over the outcome categories. There were 52 patients with NCS. Among them, 30 were of focal onset (57.7%) and 10 (5%) clearly fulfilled criteria for non-convulsive status epilepticus. There were 120 patients that had clinical seizures prior to CEEG monitoring. Among them, 36 (30%) had NCS on EEG. A total of 138 records showed RPPs, including 26 that had more than one RPP and 34 of the 52 records with NCS. A total of 78 records had only one type of RPP, including 22 (10.9%) with generalized periodic discharges (GPDs), 20 (9.9%) with lateralized periodic discharges (LPDs), 22 (10.9%) with generalized rhythmic delta activity (GRDA), and 14 (6.9%) with lateralized rhythmic delta activity (LRDA). Figure 2 shows EEG examples of LPDs and focal and generalized NCS. Sixty two patients (30.7%) out of the entire sample were deceased at 60 days after admission. This proportion was significantly higher in patients who had NCS than those who didn’t (42% vs 27%, χ ^2^ = 4.4, df=2, p=0.03) ( Table 1). The frequency of NCS etiologies in the group that was deceased at 60 days is shown in Figure 1. There was no statistically significant difference in mortality risk between the etiologies. There was no significant difference in the duration of hospital stay between those who had NCS and those who didn’t (p=0.2) ( Table 1). To analyze outcome data in relation to having RPPs, cases with RPPs but without seizures (a total of 104 cases) were considered. There was no statistically significant difference between the proportion of patients that were deceased at 60 days among those who had RPPs and the same proportion among those without relevant CEEG patterns (χ ^2^ = 0.8, df=2, p=0.2). Furthermore, there was no statistically significant difference between the two groups with regards to duration of hospitalization (χ ^2^ = 3.1, df=2, p=0.2) ( Table 1).

**Table 1.** Cross-tabulation of mortality and duration of hospital stay in relation to the presence of non-convulsive seizures (NCS) and rhythmic or periodic patterns (RPPs) on CEEG.

Variable		Mortality		Hospital Stay		
		Dead at 60 days	Alive at 60 days	Hospital stay <1 week	1 week-1month	>1month
NCS	No	40	110	44	62	44
	Row%	**26.7%**	73.3%	29.3%	41.3%	29.3%
	Col.%	64.5%	78.6%	78.6%	75.6%	68.8%
	Yes	22	30	12	20	20
	Row%	**42.3%** *	57.7%	23%	38.5%	38.5%
	Col.%	35.5%	21.4%	21.4%	24.4%	31.2%
RPPs	No	10	36	18	16	12
	Row%	21.7%	78.3%	39.1%	34.8%	26.1%
	Col.%	25%	32.7%	40.9%	25.8%	27.3%
	Yes	30	74	26	46	32
	Row%	28.8%	71.2%	25%	44.2%	30.8%
	Col.%	75%	67.3%	59.1%	74.2%	72.7%

*p<0.05.

## Discussion

The practice of using CEEG in the ICU has developed rapidly over the past decade, particularly in North America and Europe ^1, 5^. This study is one of the first to report the experience of using ICU CEEG in Saudi Arabia, a country with a rapidly developing healthcare system that faces economic constraints. The data are consistent with prior knowledge and experience from other countries that CEEG is effective in detecting NCS and other likely harmful subclinical EEG patterns ^5, 6^. This study reveals statistically significant associations between NCS and mortality, supporting the clinical gestalt of identifying and treating potentially harmful CEEG patterns (although the interpretation of this finding is limited by the inability to control for illness severity and comorbidities in this study). Having RPPs without NCS was not associated with negative outcomes in this study. Furthermore, the potential incurred excess risks of morbidity and mortality in patients with NCS and RPPs was relatively modest. Such modest increases are not likely to be of clinical significance to clinicians evaluating patient prognoses. Nonetheless, this study was a retrospective study with limitations that preclude definitive conclusions about morbidity and mortality risk magnitudes. These are better assessed by prospective studies and in well-developed CEEG programs.

Prior studies have not definitively proven that utilizing CEEG leads to better outcomes ^2, 5^. This, coupled with the significant resources required to effectively run an ICU CEEG program ^2^, may lead decision makers in developing healthcare systems to hesitate to support the development of CEEG practices and research. This study presents data from a small and developing program to demonstrate real-world effectiveness of CEEG in detecting potentially harmful electrophysiological patterns. In addition, the study highlights the uncertainties regarding the clinical significance of the prognostic information provided by CEEG. Hopefully, this should lead to further development of ICU CEEG programs with embedded prospective, patient-centered research programs. Such research should focus on how CEEG may be used effectively and optimally and how the generated data may impact clinical decisions and patient outcomes in the ICU.

This study is a retrospective analysis with limitations. Retrospective EMR data did not contain accurate information with regards to the extent and evolution of mental status changes relative to the timing of the CEEG changes, use and titration of sedatives, and other management decisions. Data concerning illness severity were also not recorded in the EMR, limiting the feasibility of controlling for illness severity and comorbidities during the analysis. As such, it cannot be ruled out that differences between groups were in part due to differences in illness severity. It was difficult to ascertain the timing of EEG initiation in relation to these dynamic variables of interest. Physicians did not follow a clear protocol when deciding the duration of the CEEG study. Furthermore, a selection bias is introduced because of the lack of EEG technologists at night. Shortages in machines and technicians may have affected the duration of CEEG monitoring, as well as treatment decisions and outcomes. Longer studies may lead to higher detection rates of relevant CEEG patterns. Although the total number of cases was 202, the number of cases in most diagnostic categories was not high enough to permit subgroup analyses. The lack of significant associations between negative outcomes and the presence of RPPs without NCS should be interpreted with caution since our data lumped all types of RPPs together and was not sufficiently powered to study each subtype of RPPs. Finally, the clinical setting of this study is that of a developing program with limited resources and must be interpreted in this context. Further studies from developing healthcare systems like Saudi Arabia’s are needed to illuminate how the practice of CEEG monitoring may be best utilized to provide clinically meaningful data while caring for patients in the ICU.

## Data availability

Open Science Framework: The yield of continuous EEG monitoring in the ICU at a tertiary care hospital in Saudi Arabia: A retrospective study. https://doi.org/10.17605/OSF.IO/Q56J3 ^4^.

This project contains all raw de-identified data associated with this study.

Data are available under the terms of the Creative Commons Zero “No rights reserved” data waiver (CC0 1.0 Public domain dedication).
