# Supplementary material for: The yield of continuous EEG monitoring in the intensive care unit at a tertiary care hospital in Saudi Arabia: A retrospective study
Source: F1000Res. 2019 Oct 18;8:663. Originally published 2019 May 15. [Version 3] doi: 10.12688/f1000research.19237.3 (PMC6807136; doi:10.12688/f1000research.19237.3)
Supplement: Peer review history

# Peer review: Reviewer response for version 3

**DOI:** 10.5256/f1000research.23073.r55417

**Published:** 2019-10-22

**Authors:** Robert C. Tasker

**Affiliations:** 1 Department of Neurology, Harvard Medical School, Boston, MA, USA

Thank you - the author has addressed my comments

I confirm that I have read this submission and believe that I have an appropriate level of expertise to confirm that it is of an acceptable scientific standard.

# Peer review: Reviewer response for version 3

**DOI:** 10.5256/f1000research.23073.r55420

**Published:** 2019-10-18

**Authors:** Peter W Kaplan

**Affiliations:** 1 Department of Neurology, Johns Hopkins Bayview Medical Center, Johns Hopkins University School of Medicine, Baltimore, MD, USA

I confirm that I have read this submission and believe that I have an appropriate level of expertise to confirm that it is of an acceptable scientific standard.

# Peer review: Reviewer response for version 2

**DOI:** 10.5256/f1000research.22446.r54710

**Published:** 2019-10-04

**Authors:** Elizabeth E Gerard, Yara Mikhaeil-Demo

**Affiliations:** 1 University of Illinois at Chicago, Chicago, USA; 2 Department of Neurology, Feinberg School of Medicine, Northwestern University (NU), Chicago, IL, 60611​, USA

This paper describes a one-year experience using continuous EEG monitoring in a developing healthcare system. The authors experience with a growing cEEG program is a valuable addition to the literature. We have some comments and questions to better understand the results described: 

**Methods:**

1. What were the inclusion and exclusion criteria? Was EEG duration less than two hours the only exclusion criteria? 
2. How frequently were studies reviewed and results communicated to treating team? It would be helpful to know the timing of when the seizure occurred on EEG and the identification of these findings on EEG and communication with the treating physician.
3. When discussing mortality and morbidity, it would be helpful to adjust for comorbid conditions and severity of underlying illness.
4. In the statistical analysis were p values adjusted for multiple comparisons?

**Results:  **

1. The author indicates a total of 202 cEEG records were identified. Are these for 202 unique patients? Did any patients have more than one cEEG study during this time? If so, were the subsequent studies excluded if the patients had more than one cEEG study during the one year period? 
2. How were the clinical diagnoses defined? For example, was “epilepsy” used only if the patient had a prior diagnosis of epilepsy or was it also used for new-onset seizures? Subcategorization of “cerebrovascular disease” would also be helpful. (Based on prior studies intracranial hemorrhage is much more likely to be associated with seizures than ischemic strokes.)
3. In addition to describing the primary diagnosis of the patients it would be helpful to know why they were connected to cEEG. What were the indications for EEG monitoring and frequency for each indication? It would be helpful to describe a table of indication for EEG monitoring and percentage for each reason (example, clinical seizure, altered mental status. abnormal movements, etc).

**Discussion:  **

1. Without adjusting for underlying diagnosis and comorbidities, it is difficult to claim that there is “a significant association between NCS and mortality”.
2. It would be helpful to provide a more detailed discussion as to why the following finding was noted “periodic or rhythmic patterns was significantly associated with longer hospital stays.”
3. In the method section, the author states: “The duration of CEEG monitoring is decided by the neurology consultation or ICU physicians.”  It appears that the longest duration for a study was 24 hours; however, as mentioned 52 patients had nonconvulsive seizures, with 10 patients fulfilling the criteria for non-convulsive status epilepticus. How were these patients monitored for response to treatment? Were NCS and NCSE successfully treated by the time of disconnect?
4. Throughout the article the authors refer to the resource requirement for CEEG monitoring. It would be interesting to know if and how resource limitations affected monitoring. What was the time from request to connection? Is there any reason to suspect this affected outcomes?  

**Figures:**  

1. Figure 2 is labeled “examples of patterns on the ictal interictal continuum;” however, clear interictal and ictal patterns are shown under this legend.  

**Tables: ** 

1. Table 1 Title has the words “on cEEG” twice.

We confirm that we have read this submission and believe that we have an appropriate level of expertise to confirm that it is of an acceptable scientific standard, however we have significant reservations, as outlined above.

# Response

**Published:** 2019-10-18

**Authors:** Haythum Tayeb

**Affiliations:** King Abdulaziz University, Saudi Arabia

Dear Dr. Gerard and Dr. Mickhaeil-Demo,

 Thank you so much for taking the time to review the second version of the manuscript and for your valuable comments that have helped me improve it. I have responded to your comments below, point by point.

 Methods:

 1. What were the inclusion and exclusion criteria? Was EEG duration less than two hours the only exclusion criteria?

 Indeed, we included all patients who had CEEG and only excluded cases that did not last for more than 2 hours.

 2. How frequently were studies reviewed and results communicated to treating team? It would be helpful to know the timing of when the seizure occurred on EEG and the identification of these findings on EEG and communication with the treating physician.

 Thank you for pointing this was not explained. I added this statement in the methods section (line 38 of the manuscript): “The electroencephalographer reviewed records at least once daily and communicated findings to the treating physicians. If seizures or other CEEG patterns of significance are revealed, physicians often decided to continue monitoring on subsequent days to guide management.”

 3. When discussing mortality and morbidity, it would be helpful to adjust for comorbid conditions and severity of underlying illness.

 Thank you for pointing out the importance of controlling for comorbid conditions and illness severity. As stated in the Methods section (line 26 of the manuscript): “The average APACHE II (Acute Physiology and Chronic Health Evaluation II) score of medical patients admitted to the ICU ranges between 10–30 on average. The scores are routinely calculated but not recorded in the electronic medical records (EMR).” As illness severity scores were not recorded in the EMR, the author had no feasible way to control for it during analysis. I added the following statement in the limitations section in the discussion (line 150 of the manuscript): “Data concerning illness severity were also not recorded in the EMR, limiting the feasibility of controlling for illness severity and comorbidities during the analysis. As such, it cannot be ruled out that differences between groups were in part due to differences in illness severity”.

 4. In the statistical analysis were p values adjusted for multiple comparisons?

 The p value was appropriate for the number of comparators and is stated for each analysis.

 Results

 1. The author indicates a total of 202 cEEG records were identified. Are these for 202 unique patients? Did any patients have more than one cEEG study during this time? If so, were the subsequent studies excluded if the patients had more than one cEEG study during the one year period?

 Thank you for pointing this needed to be clarified. I added the following to the Methods section (line 41 of the manuscript): “For the purposes of this study, records that continue for several days and those that are repeated during the same hospitalization or within 60 days of each other were considered as one recording.” This was also clarified in the results section (line 74). The outcome of mortality within 60 days and length of hospitalization were defined in relation to the cEEG records defined as such.

 2. How were the clinical diagnoses defined? For example, was “epilepsy” used only if the patient had a prior diagnosis of epilepsy or was it also used for new-onset seizures? Subcategorization of “cerebrovascular disease” would also be helpful. (Based on prior studies intracranial hemorrhage is much more likely to be associated with seizures than ischemic strokes.)

 The primary clinical diagnosis was defined by physicians taking care of the patient and entered in the EEG requisition. This is now stated results section (line 68 of the manuscript). With regards to the breakdown of cerebrovascular disease, there were 10 cases of intracranial hemorrhage. This is now stated in line 71 of the manuscript.

 3. In addition to describing the primary diagnosis of the patients it would be helpful to know why they were connected to cEEG. What were the indications for EEG monitoring and frequency for each indication? It would be helpful to describe a table of indication for EEG monitoring and percentage for each reason (example, clinical seizure, altered mental status. abnormal movements, etc).

 All patients had altered mental status. There was no reliable record of associated paroxysmal motor or non-motor events, barring the ability to provide that. This is now stated in line 31-34 of the manuscript.

 Discussion:

 1. Without adjusting for underlying diagnosis and comorbidities, it is difficult to claim that there is “a significant association between NCS and mortality”.

 I now added this statement in the Discussion section (line 129 of the manuscript): ... although the interpretation of this finding is limited by the inability to control for illness severity and comorbidities in this study.

 2. It would be helpful to provide a more detailed discussion as to why the following finding was noted “periodic or rhythmic patterns was significantly associated with longer hospital stays.”

 Following the advice of another reviewer, the manuscript now presents data of RPPs without NCS, showing no association with hospital stay. The statement in question is now removed and replaced with the following: “Having RPPs without NCS was not associated with negative outcomes in this study” (line 130 of the manuscript).

 3. In the method section, the author states: “The duration of CEEG monitoring is decided by the neurology consultation or ICU physicians.” It appears that the longest duration for a study was 24 hours; however, as mentioned 52 patients had nonconvulsive seizures, with 10 patients fulfilling the criteria for non-convulsive status epilepticus. How were these patients monitored for response to treatment? Were NCS and NCSE successfully treated by the time of disconnect?

 Thank you for pointing out the need for clarification here. This is now clarified in the methods section as follows: “Generally, physicians aim to continue monitoring for at least 24 hours in patients with altered mental status but may allow discontinuation of CEEG for clinical or practical constraints (e.g. EEG machine availability or the need to relocate a patient to conduct a procedure or test). The electroencephalographer reviewed records at least once daily and communicated findings to the treating physicians. If seizures or other CEEG patterns of significance are revealed, physicians often decided to continue monitoring on subsequent days to guide management. For the purposes of this study, records that continue for several days and those that are repeated during the same hospitalization or within 60 days of each other were considered as one recording.”

 4. Throughout the article the authors refer to the resource requirement for CEEG monitoring. It would be interesting to know if and how resource limitations affected monitoring. What was the time from request to connection? Is there any reason to suspect this affected outcomes?

 Thank you for pointing this out. I added this to the Methods section: “The time from requesting the CEEG to the beginning of the recording varied depending on availability of equipment and technologists. Generally, requests from the ICU are given priority and fulfilled within 24 hours but this is not always feasible.” I also added this to the limitations section to emphasize this point: “Shortages in machines and technicians may have affected duration of CEEG, treatment decisions and outcomes.” (line 158 of the manuscript).

 Figures:

 1. Figure 2 is labeled “examples of patterns on the ictal interictal continuum;” however, clear

 interictal and ictal patterns are shown under this legend.

 The legend is now changed to “Examples of relevant CEEG patterns from the study sample.”

 Thank you both very much for your time reviewing this manuscript and making it better.

 Sincerely,

 Haythum Tayeb

# Peer review: Reviewer response for version 2

**DOI:** 10.5256/f1000research.22446.r53969

**Published:** 2019-10-03

**Authors:** Robert C. Tasker

**Affiliations:** 1 Department of Neurology, Harvard Medical School, Boston, MA, USA

Thank you for the resubmitted report, which I have now read and find much improved. The inclusion of the flow diagram (Figure 1) is a great help. However, some additional points still need to be addressed:

**Minor:**

1. Page 3, line 4 of Results: please add "years" to the age being quoted.
2. Page 5, line 2 of Figure 2 legend: please change "uV" to the appropriate abbreviation for "microvolts".

**Major:**

1. I think that Table 1 needs to be corrected. For example, if you consider the top 6 rows related to NCS and check the numbers against the data in Figure 1 there appears to be an inconsistency. For example, row 4 tells us the number of cases with NCS as dead/alive, and by duration <1w/1w-1m/>1m. Cross-checking with Figure 1 the sub-totals for cases with NCS should add-up to 52/202, and of the 52, 22 died and 30 were alive at 60 days (22+30 = 52), which is correct. There is a problem in row 1 (the cases not exhibiting NCS). The total here should be 150 (ie, 202 minus 52). Indeed, for mortality the dead/alive (40 + 110) adds-up to 150. However, in the <1w/1w-1m/>1m three-way split, the total (44 + 60 + 44) adds-up to 148, and so 2 cases are missing. Please recheck the numbers and percentages, and the statistical tests as described in the text to make sure that everything is correct.
2. Again, Table 1, but now the bottom 6 rows related to RPPs. If you cross-check the numbers against the data in Figure 1 there appears to be some inconsistencies. For example, row 4 (row 10 in the Table) tells us the number of cases with RPPs as dead/alive, and by duration <1w/1w-1m/>1m. Cross-checking with Figure 1 the sub-total of cases with RPP should add-up to 104. however, in the dead/alive split we have 46 + 92 = 138 (so an extra 34 cases). In the <1w/1w-1m/>1m three-way split, the total (30 + 62 + 46) also adds-up 138 (again an extra 34 cases). I think, based on the data in Figure 1, the three-way split (<1w/1w-1m/>1m) should in fact be 26+46+32=104. Then, the first row of this subsection (row7 in the Table) then needs to be adjusted, and the statistics rechecked. 

Thank you for the extra work in doing this - making sure that number are accurate and consistent is always a hard task.

I confirm that I have read this submission and believe that I have an appropriate level of expertise to state that I do not consider it to be of an acceptable scientific standard, for reasons outlined above.

# Response

**Published:** 2019-10-18

**Authors:** Haythum Tayeb

**Affiliations:** King Abdulaziz University, Saudi Arabia

Dear Dr. Tasker,

 Thank you very much for re-reviewing the manuscript. I highly appreciate the time and effort you invested.

 I have made the necessary adjustments you recommended. I added “years” (page 3) and used the appropriate abbreviation for microvolts in the legend for figure 2.

 Thank you for pointing out the discrepancy between the table and the flowchart. This discrepancy in the part of the table with data regarding RPPs was because the numbers represented all cases with RPPs (including those who had NCS as well, a total of 138 cases indeed). There was also a typographical error row 1.

 Following your recommendation and realizing the importance of separating RPPs from NCS, I made the following changes:

 1. I fixed the typographical error in row 1 of the table (60 was changed to 62).

 2. I replaced the prior numbers in the last 6 rows of the table with the numbers of cases with RPPs

 without NCS. When these cases are considered, the association between RPPs and length of hospital stay drops out of significance. Accordingly, I adjusted that in the abstract, results, and

 discussion sections as well.

 3. I changed the flowchart slightly to clarify that that the 52 cases of NCS include cases that also

 had RPPs.

 Once again, thank you for your valuable time, effort, and insights.

 Sincerely,

 Haythum Tayeb

# Peer review: Reviewer response for version 2

**DOI:** 10.5256/f1000research.22446.r53968

**Published:** 2019-09-16

**Authors:** Peter W Kaplan

**Affiliations:** 1 Department of Neurology, Johns Hopkins Bayview Medical Center, Johns Hopkins University School of Medicine, Baltimore, MD, USA

I confirm that I have read this submission and believe that I have an appropriate level of expertise to confirm that it is of an acceptable scientific standard.

# Peer review: Reviewer response for version 1

**DOI:** 10.5256/f1000research.21080.r54081

**Published:** 2019-09-18

**Authors:** Elizabeth E Gerard, Yara Mikhaeil-Demo

**Affiliations:** 1 University of Illinois at Chicago, Chicago, USA; 2 Department of Neurology, Feinberg School of Medicine, Northwestern University (NU), Chicago, IL, 60611​, USA

This paper describes a one-year experience using continuous EEG monitoring in a developing healthcare system. The authors experience with a growing cEEG program is a valuable addition to the literature. We have some comments and questions to better understand the results described: 

**Methods: ** 

1. What were the inclusions and exclusions criteria? Was EEG duration less than two hours the only exclusion criteria?
2. How frequently were studies reviewed and results communicated to treating team? It would be helpful to know the timing of when the seizure occurred on EEG and the identification of these findings on EEG and communication with the treating physician.
3. When discussing mortality and morbidity, it would be helpful to adjust for comorbid conditions and severity of underlying illness.
4. In the statistical analysis were p values adjusted for multiple comparisons? 

**Results: ** 

1. The author indicates a total of 202 cEEG records were identified. Are these for 202 unique patients? Did any patients have more than one cEEG study during this time? If so, were the subsequent studies excluded if the patients had more than one cEEG study during the one-year period?
2. How were the clinical diagnoses defined? For example, was “epilepsy” used only if the patient had a prior diagnosis of epilepsy, or was it also used for new-onset seizures? Subcategorization of “cerebrovascular disease” would also be helpful. (Based on prior studies intracranial hemorrhage is much more likely to be associated with seizures than ischemic stroke.)
3. For Table 2, did any patients have more than one of the periodic patterns? Did patients have NCS and periodic patterns? Why is NCSE not listed in the table?
4. It would be interesting to look at the diagnoses associated with NCS, NCSE and periodic patterns.
5. In addition to describing the primary diagnosis of the patients it would be helpful to know why they were connected to cEEG. What were the indications for EEG monitoring and frequency for each indication? It would be helpful to describe a table of indication for EEG monitoring and percentage for each reason (example, clinical seizure, altered mental status. abnormal movements, etc). 

**Discussion:  **

1. The author states: “The data are consistent with prior knowledge and experience from other countries that CEEG is effective in detecting NCS and other likely harmful subclinical EEG patterns on the ictal-interictal continuum.” This paper does not define nor discuss the ictal-interictal continuum.
2. The discussion should compare the findings at the current center to prior publications on the yield of CEEG.
3. Without adjusting for underlying diagnosis and comorbidities, it is difficult to claim that there is “a significant association between NCS and mortality.”
4. It would be helpful to provide a more detailed discussion as to why the following finding was noted “periodic or rhythmic patterns was significantly associated with longer hospital stays.”
5. In the method section, the author states: “The duration of CEEG monitoring is decided by the neurology consultation or ICU physicians.”  It appears that the longest duration for a study was 24 hours; however, as mentioned 52 patients had nonconvulsive seizures, with 10 patients fulfilling the criteria for non-convulsive status epilepticus. How were these patients monitored for response to treatment? Were NCS and NCSE successfully treated by the time of disconnect?
6. Throughout the article the authors refer to the resource requirement for CEEG monitoring. It would be interesting to know if and how resource limitations affected monitoring. What was the time from request to connection? Is there any reason to suspect this affected outcomes?

We confirm that we have read this submission and believe that we have an appropriate level of expertise to confirm that it is of an acceptable scientific standard, however we have significant reservations, as outlined above.

# Peer review: Reviewer response for version 1

**DOI:** 10.5256/f1000research.21080.r51103

**Published:** 2019-07-23

**Authors:** Robert C. Tasker

**Affiliations:** 1 Department of Neurology, Harvard Medical School, Boston, MA, USA

The author has described a practice of continuous EEG monitoring in an intensive care unit in a University Hospital in Jeddah, Saudi Arabia.

 I have a number of comments that will help readers better understand what these data mean, and how they might reflect on the implications for ICU practice.

**Method** (page 3)

1. In the first paragraph (line 8) the author describes "CEEG are requested by ICU physician or neurologists according to the clinical needs". In order to understand the significance of some of the analyses provided, it would be helpful to know what were the inclusion criteria, or the 'standard operating procedure' for the ICU. For example, were all patients comatose, and the clinician was unable to assess a clinical response?
2. In the first paragraph (lines 13-15) the authors describes "The duration of CEEG monitoring is decided by the neurology consultation or ICU physicians":
  - In order to understand the significance of some of the analyses later in the manuscript, it would be helpful to know when CEEG was started and finished in relation to admission and discharge/death.
  - It would also be helpful to know the timing of when NCS occurred.
3. Since this population has been gathered from ICU admissions, it would be helpful to have some description of ICU severity-of-illness, according to the risk-adjustment score used by the unit. The examination of any illness feature that might be associated with death needs to be adjusted for severity of illness. There were 62 observed deaths in 200 cases - what was the expected number of deaths from the admission data? 

**Results** (pages 3 and 4)

1. The presentation of findings in the Results section and the Abstract is a little confusing:
  - The author has a starting population of 202 patients undergoing CEEG monitoring. Then, the denominators being used in the data summaries are n=120, and n=200. I think that a flow chart would help here.
2. Table 2 can be summarized as text, which will make room for a flow-diagram and better review of the data presented in Table 3.
3. Table 3 needs some attention to data accuracy. For example, in Row 2 (Hospital stay data) the percentages add up to 101%, which is just a rounding error. In Row 3 (Hospital stay data) the percentages add up to 99%.
4. Yes, the author has found some associations with death, and length of stay, but does the information gained from CEEG have the potential to help with decision making? For example, if we imagined that CEEG was a 'diagnostic test', then:
  - The pre-CEEG probability of death in this population was 31%. The finding of EEG-seizures could change your pre-test to a post-test probability of death of 39%. I don't think that physicians would find this a helpful piece of information. That is, it is not about death, rather it is something we should use to identify a need for treatment, for example.
  - Similarly, the pre-CEEG probability of hospital stay >1 month was 32%. The finding of periodic or rhythmic patterns could change the pre-test to post-test probability of hospital stay >1 month to 34%. Again, I don't think that physicians would find this a helpful piece of information. 
  - Obviously, both of these points would need to be tested and validated in a prospective study (as well as knowing details of when these CEEG features were found), but it may lead to a change in emphasis in the first paragraph of the Discussion.

**Discussion** (pages 3 and 4)

 Based on the above results, one could conclude that identifying EEG seizures on CEEG does not provide a significant 'quantum' in prognostic evidence. Similarly, identifying periodic or rhythmic patterns on CEEG does not provide a significant 'quantum' in identifying cases that will be likely be in-hospital >1 month.

 I am wondering whether the focus should be on 'what are we doing the CEEG': surely it is to identify something that we can/should treat; and, to identify something that will help us determine that we no longer need to do CEEG.

I confirm that I have read this submission and believe that I have an appropriate level of expertise to state that I do not consider it to be of an acceptable scientific standard, for reasons outlined above.

# Response

**Published:** 2019-08-25

**Authors:** Haythum Tayeb

**Affiliations:** King Abdulaziz University, Saudi Arabia

Dear Dr. Tasker,

Thank you very much for your time and detailed review of my manuscript entitled: “The yield of continuous EEG monitoring in the intensive care unit at a tertiary care hospital in Saudi Arabia: A retrospective study”. I value your insights. I have submitted a new version of this manuscript that took into consideration all of your comments and followed your suggestions as well as the other peer review I received. I respond to your comments below as well:

1. it would be helpful to know what were the inclusion criteria, or the 'standard operating procedure' for the ICU.

I added this statement in the Data Gathering section of the new version of the article to clarify the standard practice of the physicians when it comes to ordering, starting and stopping CEEG: “ICU physicians or neurologists request CEEG to search for NCS or patterns on the ictal-interictal continuum when critically ill patients have a disturbance in the level of consciousness that is unexplained by apparent underlying neurological or medical conditions. CEEG is initiated and stopped based on the clinical judgment of the treating teams. Generally, physicians aim to continue monitoring for 24 hours in patients with altered mental status but may allow discontinuation of CEEG for clinical or practical constraints (e.g. EEG machine availability or the need to relocate the patients).”

In this developing program, there is no hospital protocol with regards to patient selection, CEEG initiation or termination. This is entirely left to the discretion of the treating physicians and is typically reached through discussion and consultation between neurology and ICU. Generally, all patients with unexplained altered mental status were included. It was difficult to ascertain from the EMR documentation how the mental status evolved during the time of the CEEG, unfortunately. This is a limitation I state in the Discussion section.

2. In order to understand the significance of some of the analyses later in the manuscript, it would be helpful to know when CEEG was started and finished in relation to admission and discharge/death. It would also be helpful to know the timing of when NCS occurred.

It was difficult to determine with consistency retrospectively from EMR how the duration of monitoring was decided and when the seizures occurred relative to the outcomes of interest. I agree that this is a limitation and would be best addressed in a prospective study. I added this to the new version of the article in the discussion section: “Retrospective EMR data did not contain accurate information with regards to the extent and evolution of mental status changes relative to the timing of the CEEG, use and titration of sedatives, and other management decisions. It was difficult to ascertain the timing of EEG initiation in relation to these dynamic variables of interest. Physicians did not follow a clear protocol when deciding the duration of the CEEG study.”

3. Since this population has been gathered from ICU admissions, it would be helpful to have some description of ICU severity-of-illness, according to the risk-adjustment score used by the unit. The examination of any illness feature that might be associated with death needs to be adjusted for severity of illness. There were 62 observed deaths in 200 cases - what was the expected number of deaths from the admission data? 

Thank you for pointing this out. I added this to the methods section: “The average APACHE II (Acute Physiology and Chronic Health Evaluation II) score of medical patients admitted to the ICU ranges between 10-30 on average. The scores are routinely calculated but not recorded in the electronic medical records (EMR).” This was a limitation of the study and we could not remedy this by calculating scores retrospectively.

4. The author has a starting population of 202 patients undergoing CEEG monitoring. Then, the denominators being used in the data summaries are n=120, and n=200. I think that a flow chart would help here.

I added a flow chart. Thank you for suggesting it. n=120 is the number of patients who had clinical seizures prior to CEEG initiation. This is a group of interest since data have previously shown a ~30% risk of NCS. We replicated this. I removed n=200 from the prior reporting of the Chi-square to avoid ambiguity. Thank you.

5. Table 2 can be summarized as text, which will make room for a flow-diagram and better review of the data presented in Table 3.

Done. I expanded table 1 (formerly table 3) by adding row and column percentages.

6. Table 3 needs some attention to data accuracy. 

Thank you for pointing this out. I scrutinized the data and ensured accuracy. I fixed several coding errors that altered the proportions slightly without altering the overall outlook of the results as well.

7. Does the information gained from CEEG have the potential to help with decision making?

Thank you very much for highlighting this issue. To address it, I reported the “pre-test” proportions to show that the “post-test” proportions are not of large magnitudes. I also added row and column percentages in table 1.

8. I am wondering whether the focus should be on 'what are we doing the CEEG': surely it is to identify something that we can/should treat; and, to identify something that will help us determine that we no longer need to do CEEG.

Thank you for this insight. I altered the discussion to reflect the uncertainty about the clinical meaningfulness of the prognostic information and highlight the need for prospective studies to address the important questions. This is from the new version of the manuscript: “This study reveals statistically significant associations between NCS and mortality and between RPPs and longer hospital stays, supporting the clinical gestalt of identifying and treating potentially harmful CEEG patterns is worthwhile. However, the incurred excess risks of morbidity and mortality in patients with NCS or RPPs was relatively modest. Such modest increases are not likely to be of clinical significance to clinicians evaluating patient prognoses. Nonetheless, this study was a retrospective study with limitations that preclude definitive conclusions about morbidity and mortality risk magnitudes, which are better assessed with prospective studies and in well-developed CEEG programs.

Prior studies have not definitively proven that utilizing CEEG leads to better outcomes ^2^^, ^^5^ . This, coupled with the significant resources required to effectively run an ICU CEEG program ^2^ , may lead decision-makers in developing healthcare systems to hesitate to support the development of CEEG practices and research. This study presents data from a small and developing program to demonstrate the real-world effectiveness of CEEG in detecting potentially harmful electrophysiological patterns. In addition, the study highlights the uncertainties regarding the clinical significance of the prognostic information provided by CEEG. Hopefully, this should lead to further development of ICU CEEG programs with embedded prospective, patient-centered research programs. Such research should focus on how CEEG may be used effectively and optimally and how the generated data may impact clinical decisions and patient outcomes in the ICU.”

Once again, thank you very much for your thorough review of this manuscript and your valuable insights. Much appreciated.

Sincerely,

Haythum Tayeb

# Peer review: Reviewer response for version 1

**DOI:** 10.5256/f1000research.21080.r48822

**Published:** 2019-05-23

**Authors:** Peter W Kaplan

**Affiliations:** 1 Department of Neurology, Johns Hopkins Bayview Medical Center, Johns Hopkins University School of Medicine, Baltimore, MD, USA

How many of the NCSE were focal versus generalized? 

 Did not the mortality vary with etiology of NCSE and PDs?

 I would add in the final section on limitations of the study that there is perforce selection bias as the EEG techs were not available during certain periods (if I remember, overnight and other times) and this should be stated. 

 Possibly an EEG example of NCSE should be provided.

I confirm that I have read this submission and believe that I have an appropriate level of expertise to confirm that it is of an acceptable scientific standard.

# Response

**Published:** 2019-08-25

**Authors:** Haythum Tayeb

**Affiliations:** King Abdulaziz University, Saudi Arabia

Dear Dr. Kaplan,

Thank you very much for kindly reviewing my manuscript entitled: “The yield of continuous EEG monitoring in the intensive care unit at a tertiary care hospital in Saudi Arabia: A retrospective study”. I value your insights. I have submitted a new version of this manuscript that took in consideration your comments and followed your suggestions as well as the other peer review I received. I briefly respond to your comments below as well:

1. How many of the NCSE were focal versus generalized? 

I included in the new version of the article that "There were 52 patients with NCS. Among them, 30 were of focal onset (57.7%)".

2. Did not the mortality vary with etiology of NCSE and PDs?

The frequency of NCS etiologies in the group that was deceased at 60 days is now shown in a flow chart figure (figure 1). There was no statistically significant difference in mortality risk between the etiologies. Unfortunately, the number of cases in most subgroups was too small to permit subgroup analysis. I addressed this in the new version of the manuscript.

3. I would add in the final section on limitations of the study that there is perforce selection bias as the EEG techs were not available during certain periods (if I remember, overnight and other times) and this should be stated. 

Added. 

4. Possibly an EEG example of NCSE should be provided.

Added. 

Once again, thank very much for your time and insights.

Sincerely,

Haythum Tayeb
